# Standardization and accuracy of race and ethnicity data: Equity implications for medical AI

**DOI:** 10.1371/journal.pdig.0000807

**Published:** 2025-05-29

**Authors:** Alexandra Tsalidis, Lakshmi Bharadwaj, Francis X. Shen

**Affiliations:** 1 Future of Life Institute, Brussels, Belgium; 2 Laboratory of Biochemical Pharmacology, Emory University School of Medicine, Atlanta, Georgia, United States of America; 3 Department of Pediatrics, Emory University School of Medicine, Atlanta, Georgia, United States of America; 4 University of Minnesota Law School, Minneapolis, Minnesota, United States of America; 5 Massachusetts General Hospital Center for Law, Brain & Behavior, Boston, Massachusetts, United States of America; 6 Department of Neurosurgery, Dana Foundation Neurotech Justice Accelerator at Mass General Brigham, Boston, Massachusetts, United States of America,; 7 Center for Bioethics, Harvard Medical School, Boston, Massachusetts, United States of America; Emory University, UNITED STATES OF AMERICA

## Abstract

The rapid integration of artificial intelligence (AI) into healthcare has raised many concerns about race bias in AI models. Yet, overlooked in this dialogue is the lack of quality control for the accuracy of patient race and ethnicity (r/e) data in electronic health records (EHR). This article critically examines the factors driving inaccurate and unrepresentative r/e datasets. These include conceptual uncertainties about how to categorize races and ethnicity, shortcomings in data collection practices, EHR standards, and the misclassification of patients’ race or ethnicity. To address these challenges, we propose a two-pronged action plan. First, we present a set of best practices for healthcare systems and medical AI researchers to improve r/e data accuracy. Second, we call for developers of medical AI models to transparently warrant the quality of their r/e data. Given the ethical and scientific imperatives of ensuring high-quality r/e data in AI-driven healthcare, we argue that these steps should be taken immediately.

## Author summary

Healthcare systems are increasingly using artificial intelligence (AI) to improve clinical care in various settings such as hospitals and patient care facilities. In this paper, we discuss how these AI systems may be trained using inaccurate and incomplete patient race and ethnicity data. We identify several key issues underlying this data quality problem: the conceptual challenges in defining race and ethnicity categories, inconsistent data collection practices across healthcare facilities, and frequent errors in classifying patients. These problems create unreliable training data that undermines efforts to avoid and correct biases within these medical AI tools. To address these challenges, we propose two practical solutions. First, hospitals should adopt best practices for collecting race and ethnicity information, including patient self-reporting, staff training, and transparent processes. Second, developers of medical AI should be required to disclose the quality and sources of the demographic data used to train their models. Our work emphasizes that discussions about fairness in medical AI must include attention to the quality of race and ethnicity data. As these technologies become more widespread in healthcare, ensuring they work effectively for all patients requires addressing these fundamental data issues.

## I. Introduction

Artificial intelligence (AI) is being rapidly deployed in many healthcare contexts, with many of these systems relying on individual-level patient data from electronic health records (EHR). There are widespread concerns about potential race bias in such heath care AI models, including critiques raised by many researchers [1–3], the American Civil Liberties Union (ACLU) [4], and the World Health Organization [5]. The E.U. AI Act mandates that the datasets used to train, validate, and test high-risk AI systems be as representative, error-free, and complete as possible (Art. 10.3).

**Fig 1:**
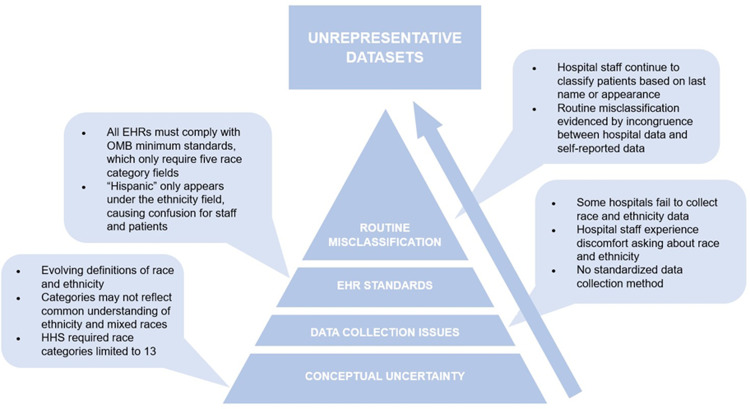
Tracing the steps that lead to unrepresentative race and ethnicity datasets.

Yet, overlooked in this evolving dialogue is that without accurate and standardized patient race and ethnicity (r/e) data, both the AI models *and* the methods proposed to identify and address r/e bias will fail to deliver on their promise. The road to unrepresentative r/e datasets is paved with procedural mistakes and conceptual uncertainty (see Fig 1 below). As noted in Fig 1, challenges begin with the conceptual question of how, theoretically, to define “race” and “ethnicity”. Even once definitions are in place, hospitals are inconsistent in their collection of r/e data. Variation in how this data is entered into EHR systems adds further complexity, as staff may misclassify patient race. In the end, these compounded issues lead to unrepresentative datasets, with the extent of the problem remaining uncertain due to inaccuracy and gaps in r/e data collection and reporting.

An “ethical AI” strategy involving r/e data analysis can only work if (1) the r/e data is being accurately recorded at each site, and (2) the data can be harmonized across the different r/e categories used by each site. Our analysis presented below finds that these remain big “ifs”.

Three decades of scholarship in health disparities research reveals both conceptual and practical challenges in ensuring accuracy for r/e data in the EHR [6]. A 2023 systematic review of 56 datasets assessing the availability and accuracy of patient r/e data found that “EHRs often had missing and/or inaccurate data on race/ethnicity” and that these inaccuracies were most acute for non-white populations [7]. The old axiom—“garbage in, garbage out”—applies. If the r/e data on which medical AI is trained cannot be trusted, then neither can the resulting models nor the mitigating strategies that rely on analyses of said data.

This raises an even more fundamental question: should biomedical research and clinical practice incorporate r/e variables at all, as distinct from utilizing measures of racism, socioeconomic status, and ancestry [8,9]? We do not advocate for or against using r/e data in AI models: whether to use r/e variables is a complex decision, and we encourage researchers to follow the excellent guidance from the National Academies of Sciences, Engineering, and Medicine on how to weigh the competing factors [9]. In some instances, after careful consideration, researchers may conclude that it is ethical and relevant to use r/e data. For example, in a study on AI models predicting glaucoma progression, incorporating r/e data improved fairness metrics like equalized odds when tested on external populations [10]. Moreover, developers may use r/e data to evaluate models post training by identifying disparities in performance across demographic groups.

Notwithstanding the importance of these broader questions, our focus is on what happens after the decision has been made to utilize r/e data. We propose two solutions to the issues engendered by this stage of the AI development process. First, we provide a quality control (QC) roadmap for healthcare systems to improve the accuracy of their r/e data for medical AI. Second, we argue that all medical AI model developers should warrant the quality (or lack thereof) of the r/e data they use.

## II. The challenge of standardizing r/e categories while federal standards are in flux

A prerequisite for standardizing r/e data across sites is clearly defining the r/e categories themselves. This is no simple task given that race and ethnicity are social constructs – they have always been fluid categories and will likely continue to evolve. At present, the U.S. Government is revisiting the most widely used categories in U.S. biomedical research and clinical practice.

### Revisiting OMB standards

In 1997, the Office of Management and Budget (OMB) established standardized questions on r/e that federal agencies and recipients of federal funds were obligated to report data on. This move was motivated by a need to enforce civil rights laws, especially by monitoring equal access to housing, education, and employment for “population groups that historically had experienced discrimination and differential treatment” [11]. In March 2024, after nearly two years of consultation and input, the OMB published revisions to Statistical Policy Directive No. 15 [12]. The new directive notes that “the race and ethnicity categories set forth are sociopolitical constructs and are not an attempt to define race and ethnicity biologically or genetically” [12]. It is beyond the scope of this article to examine the new OMB directive in detail, but we address it further below.

Prior to the 2024 revisions, the OMB standards had been comprised of two ethnicity categories (“Hispanic or Latino” and “Not Hispanic or Latino”) and five race categories. Section 3101 of the Public Health Service Act also requires any federally conducted or supported healthcare or public health program, activity, or survey to collect and report data using the Department of Health & Human Services (HHS) standards [13]. The HHS standards broke down the OMB’s five race categories into 13 subcategories, and the two ethnicity categories into four [14]. Individual hospitals can create more granular r/e categories, but these must roll up into the OMB standards. While hospitals not receiving federal funding will be exempt from these standards, professional associations like the American Hospital Association recommend the collection of r/e data [15].

Others have sought to standardize r/e data collection, while still conforming to the OMB standards. For example, the Observational Outcomes Partnership (OMOP) established the Common Data Model (CDM), “an open community data standard, designed to standardize the structure and content of observational data” [16]. Although there has been significant internal debate on how race and ethnicity should be standardized [17], the CDM currently follows OMB standards, with the “Race” field containing dozens of concepts, while the “Ethnicity” field contains two [18].

The new OMB standards emerged from years of debate. In June of 2022, the Chief Statistician of the U.S. announced that his office would be reviewing and revising current OMB standards on r/e data [19]. This move was in recognition of (1) increasing racial and ethnic diversity; (2) the growing number of people identifying as more than one race or ethnicity; and (3) changing immigration and migration patterns. Following OMB’s request for comment on some initial proposals, a debate about r/e data categories has resurfaced, with some stakeholders advocating for the r/e questions to be combined, given evidence that many respondents view race and ethnicity as similar or the same [20]. However, other presenters raised concerns that combining race and ethnicity might lead to erasure within racially and ethnically diverse communities. For example, Afro-Latino patients could feel compelled to choose between the ‘Hispanic or Latino’ and the ‘Black or African American’ categories, when they would otherwise have categorized themselves as both [20].

### Conceptual challenges

Developing categories for r/e is an epistemically challenging undertaking with broad social consequences for medical research. To take just one example, scholars disagree on whether the ‘one drop rule’ (the idea that anyone with a Black ancestor is considered Black) is a reductionist approach and ignores the complexities of identity and culture, reinforcing negative stereotypes like that of racial purity [21]. Relatedly, some have advocated for using ‘multiracial’ as an r/e category [22], while others oppose this designation [23]. Moreover, there is discourse on whether r/e categories should be used at all, or whether they should be replaced by ancestry in biomedical research and clinical practice [24]. R/e categories are essential for understanding health disparities and developing targeted interventions, but there are those who criticize their oversimplification and failure to capture the complexity of identity and culture [25,26].

These historical debates were considered in the National Academies of Sciences, Engineering, and Medicine (NASEM) report on the Use of Race, Ethnicity, and Ancestry as Population Descriptors in Genomics Research [27]. The standardization of r/e categories for use in biomedical AI modeling should be guided by these particularly relevant recommendations:

- “[R]esearchers should avoid typological thinking, including the assumption and implication of hierarchy, homogeneity, distinct categories, or stability over time of the groups.”
- “Researchers, as well as those who draw on their findings, should be attentive to the connotations and impacts of the terminology they use to label groups.”
- “For each descriptor selected, labels should be applied consistently to all participants.”
- “Researchers should disclose the process by which they selected and assigned group labels…”

## III. The challenge of accuracy in r/e data collection

Beyond these conceptual issues, the challenge of matching a patient to the right category remains. The same challenges have long applied to sexual orientation and gender identity and expression (SOGIE) data collection [28]. Research on health disparities has repeatedly revealed significant methodological problems and systemic issues in r/e data collection, including clerks determining a patient’s race or ethnicity based on observation, often using last name or appearance [29]. One analysis comparing administrative r/e data to patients’ self-reported data identified low agreement rates for certain races including Pacific Islanders, Asians, and Native Americans [30]. Another study revealed similar results for patients who were admitted to two different hospitals [31]. If r/e data collection was accurate, these patients should have been categorized as the same race across the two hospitals. Yet, while White and Black patients were usually classified under the same racial category in both hospitals, patients in all other categories had very low reliability coefficients. More recent studies have continued raising doubts about the accuracy of r/e categorization, especially for marginalized populations. A smaller-scale survey of patients at two New York City clinics found that 33% of respondents at Clinic A and 22% of respondents at Clinic B self-identified in a different manner than the race or ethnicity they were recorded as in the clinic registration database [32]. Around a quarter of patient records in two large observational health databases in the U.S. contained “uninformative” r/e data (either categorized as “Unknown” or “Declined to Answer”) [33]. Moreover, 57.9% of the 2.5 million patients served by a New York City healthcare system did not have a race or ethnicity identified in their EHRs [33]. At the same time, 66.5% of patients who recorded their own race and ethnicity selected different categories than their EHR [33].

While certain best practices for collecting r/e data have emerged (including the NIH All of Us Research Program’s Participant Provided Information (PPI) form), obtaining that data in the biomedical context, especially in critical care situations, is challenging. An additional challenge of accurately capturing patient r/e data is the general reluctance of hospital staff to ask patients to self-identify. It has been found that hospital staff, in particular clerks and administrators, feel discomfort asking these questions which may be interpreted as intrusive or offensive [34,35]. At the same time, one study showed that 28% of respondents were uncomfortable sharing r/e information with a clerk or administrator [34]. Recent scholarship by Owosela et al. (2024) confirms that these issues persist [6]. In their review of the accuracy of r/e data in EHRs, Owosela et al. find that inaccuracies in r/e persist, owing in part to the limitations of self-reporting and a lack of standardization. Their recommendations for improved self-reporting mechanisms and standardization are consistent with our analysis below.

Misclassification of patient r/e is directly related to the standardization and categorization challenges discussed above. Both patients and hospital staff may be confused or frustrated by the options made available to them on intake forms and in EHR drop-down menus [27]. Our experience with Bridge2AI: Patient-Focused Collaborative Hospital Repository Uniting Standards (CHoRUS) for Equitable AI, an NIH project developing an AI-ready dataset from more than 100,000 critically ill patients’ EHR data, illuminates the urgency of the problem. While all sites in the project adhered to the two standard OMB ethnicity options, some provided over 100 subcategories. Some of the sites address the challenge of multiracial patients by having them select a ‘multiracial’ option, while others have patients check off all applicable r/e options. For medical AI, the implication is that deployment and development across sites may be significantly hampered if the sites are not consistent and accurate in how they classify patient r/e.

These inaccuracies in r/e data collection have potentially pernicious implications for medical AI development. To illustrate, a recent review examining the performance of AI models in treating cardiovascular diseases found racial disparities and concluded that it is essential to identify strategies to limit and mitigate bias during each step of the AI development pipeline [36]. Similar issues have been raised around the deployment of AI models in dermatology [37], radiology [38], and other medical fields [39]. Given these credible concerns about race bias in medical AI systems, it follows that inaccurate r/e demographic data could amplify health inequities by preventing new AI tools from working equally well across all r/e groups.

## IV. Toward solutions

To address these issues, we propose two solutions: (1) hospital systems should adopt best practices in r/e data Quality Control (QC), and (2) researchers and developers creating medical AI models should explicitly warrant the quality of their r/e data.

### Quality control

Based on two decades of research on optimizing the collection of r/e data in hospital contexts, recommendations for best practices have emerged. In Box 1, we identify several of these best practices that are germane to the issues we raise here (Box 1). The first step is to prioritize this problem on an institutional level and develop a plan for improving r/e data classification accuracy. This plan should be patient-centered, facilitating patient autonomy over how their r/e data is reported and then shared. Patients should also be given a clear rationale for why their r/e information is being requested.

Given the diversity of self-identification preferences and significant cross-cultural and cross-national differences in how r/e is conceptualized, we recognize that it is impossible to create r/e categories that perfectly reflect the sociodemographic groups with which individuals identify. But if the representativeness of AI datasets is to be evaluated, it follows that a categorization scheme must be deployed. Our contention is that in refining those categories institutions should continue to develop more granular categories reflecting the diversity of their unique communities. Precisely because these categories are challenging to define, and vary across cultures, it is important for institutional policymakers to listen to concerns from members of their communities.

Our proposed solution seeks to reconcile the tension between allowing for flexibility in patient identification and facilitating cross-institutional comparisons via standardization. A lack of standardization, both in defining categories and developing procedures for operationalizing them, is at the core of the problems we have identified above (see Section III). In the absence of uniform guidelines for collecting and categorizing r/e, different healthcare institutions may use divergent approaches, leading to incomparable datasets that cannot be meaningfully merged or analyzed at a broader scale. Without a standardized approach to measuring and reporting r/e in AI healthcare training datasets, we would expect to see significant variations across institutions in the quality of r/e data. This variation would contribute to the perennial problem of aggregating across datasets from different hospitals [40]. Using fragmented datasets may also impede efforts to monitor and evaluate AI systems for bias. If standardization across major systems is not practical, then at a minimum, institutions should be transparent about their methods and ensure that however they have defined their categories, they are accurate and consistent in applying them. This transparency and granular data could facilitate standardization across institutions, even when those institutions adopt different categories.

**Box 1. *Best Practices for Obtaining Patients’ Race & Ethnicity Data.***

**Prioritize and Plan:** Make improving self-reported r/e data an organizational priority using intentional policies, procedures, and training [41].

**Self-Report, if Possible Before the Visit:** Empower patients by inviting them to self-report r/e information, including through pre-visit questionnaires [42,43].

**Follow-up:** If patient r/e data is not collected before or during the first visit, ensure that there is a personalized follow-up [44].

**Provide Rationale:** When inviting patients to share their r/e information, explain the rationale, e.g., that the hospital will use this data for quality monitoring and to improve patient care [27].

**Improved Training:** Ensure that all frontline staff who will interact with patients regarding r/e data collection have undergone appropriate training [42]. The use of scripts can be helpful in ensuring sensitivity when encouraging patients to provide their r/e data [41,43].

**Team Effort:** If a patient is uncomfortable speaking with the administrator or clerk about their r/e data, involve a doctor or nurse as this may increase comfort levels [34]. Multiple stakeholders should be responsible for the collection of this data, not just one [45].

**Electronic Health Record Fields:** Self-identifying through a paper-based form can be daunting when there is a long list of r/e labels. Online forms allowing for keystroke recognition can increase the number of r/e options used to populate fields. [27]

**Maintain Flexibility:** Hospitals should remain flexible given that the sociodemographic makeup of the communities they serve will inevitably change. Having an annual review of current census data can help ensure that an appropriate amount of granularity is included in the r/e options provided to patients [27]. The ELSIhub Collection on race and population identifiers provides a useful resource to stay up to date [46].

Addressing the practical challenges involved in implementing these best practices will need to be a priority if institutions wish to bring about real change in the quality of the r/e data they generate. We identify three practical challenges to prioritize. The first is that we are not aware of a publicly available training program that can be readily imported to address r/e data collection issues. An initial step will be investment in a short training module. The second challenge, related to the first, is that r/e data is likely to be collected by several different types of staff members, including those who admit patients, clinical staff who treat patients, and IT staff who prepare data files and link r/e data from one system to another. It is therefore important for the training module to be completed by all who may be initially collecting, reviewing, and archiving r/e data. The third challenge is that developing a training module and implementing it will face competing priorities in terms of resource allocation. We believe that investing in the implementation of these best practices is likely to yield outsized benefits in the longer term by increasing patient trust and unlocking the full potential of AI systems to improve health outcomes. An incremental, iterative approach can also help alleviate some of these challenges, as pilot programs will enable hospitals to refine workflows, train staff, and measure outcomes before scaling up across hospital systems.

### Warranting data quality

Leading AI ethics frameworks stress the importance of transparency and sharing with end users the contents of the black box supporting medical decision-making [47]. One promising strategy to promote transparency is the use of labeling for AI-based medical devices [48]. We advocate for a label on medical AI software which acknowledges data quality limitations and discloses the data collection processes used for the r/e datasets on which the models were created.

We suggest that this disclaimer could take one of the following forms:

The models reported in this analysis and/or implemented in this tool were trained with data that included race and ethnicity (r/e) descriptors for individuals. The individualized r/e data were derived from [*insert data source*].

Option #1 (preferred, if data collection methods are known): This individualized r/e data were collected in the following way: [*describe r/e collection methods*].

Option #2 (necessary if data collection methods are not known to those developing and implementing the AI models): We are not aware of how this individualized r/e data were collected, and therefore cannot warranty the quality of the r/e data used in these models.

This disclaimer could be added at minimal time cost. The collection methods that could be specified include patient self-reporting through pre-appointment online forms. Such disclaimers would serve the ethical priority of transparency in AI modeling and the scientific priority of ensuring that AI models are built on high quality data—and if not, making everyone aware of this deficiency.

We recognize that the entities and individuals developing medical AI models will often be distinct from those who are gathering demographic information. Inspired by the longstanding practice of “ethical sourcing” of materials in the field of corporate social responsibility [49], our approach places the onus on AI developers to use reasonable efforts to identify the source(s) and quality of the r/e data they deploy in their models. For example, missing r/e data is regularly imputed in healthcare datasets [50–52]. Developers should be aware of when the r/e data they are using has been imputed and should communicate this to those who use their AI systems. AI developers often work with multiple datasets to train their AI model. In such instances, we believe that developers should disclose the collection methodologies for each dataset that was utilized in the development of the medical AI model, especially when the r/e datasets differ in terms of quality.

Our approach is forward-looking and intended to spur systemic changes in r/e data collection practices. We anticipate that in the near term, many AI developers will need to select Option #2 because they will not know how the r/e data in their models was generated. This would be true, for instance, for those AI developers who are working with historical datasets. However, we also anticipate that precisely because they are initially scarcer, AI models that can provide Option #1 explanations will be more highly valued. For example, a firm could differentiate itself from competitors by pointing to its use of gold-standard r/e data. Relatedly, as medical AI faces increasing regulatory scrutiny, markets may favor those model developers who can offer an Option #1 explanation. Regulatory bodies such as the U.S. Food and Drug Administration and the E.U.’s AI Office will be actively monitoring the potential for bias in medical AI systems. In the case of the E.U. AI Act, high-risk AI systems must be trained, validated, and tested with data that meet quality criteria, which include “data collection processes and the origin of data” (Article 10(2)(b)). The path forward to more accurate r/e data in AI models may be slow, but we believe that without data quality disclaimers progress will be even slower.

## V. Conclusion

Our argument is straightforward: if medical AI models are going to utilize r/e data, that data should be of high quality. We have laid out concerns about r/e data quality and proposed high-level guidance for improving and maintaining that quality. Hospitals are the keepers of EHR data and thus are best positioned to implement our recommended QC measures. Nevertheless, researchers and developers who are creating AI models for biomedical use have a parallel ethical responsibility to know where their r/e datasets are coming from and to be transparent about the possible deficiencies of those datasets on which their models are built. For norms to change, professional societies, funders, journal editors, and peer reviewers will need to demand better r/e data accountability. A productive next step would be to convene these stakeholders, along with patient advocates, to further develop the initial solutions we have proposed.
